# Fucoidan from *Sargassum autumnale* Inhibits Potential Inflammatory Responses via NF-κB and MAPK Pathway Suppression in Lipopolysaccharide-Induced RAW 264.7 Macrophages

**DOI:** 10.3390/md21070374

**Published:** 2023-06-25

**Authors:** N. M. Liyanage, Hyo-Geun Lee, D. P. Nagahawatta, H. H. A. C. K. Jayawardhana, Kyung-Mo Song, Yun-Sang Choi, You-Jin Jeon, Min-Cheol Kang

**Affiliations:** 1 Department of Marine Life Sciences, Jeju National University, Jeju 63243, Republic of Korea; liyanagenm@jejunu.ac.kr (N.M.L.); hyogeunlee92@jejunu.ac.kr (H.-G.L.); pramuditha1992@jejunu.ac.kr (D.P.N.); chathuri.k.j@stu.jejunu.ac.kr (H.H.A.C.K.J.); 2 Research Group of Process Engineering, Korea Food Research Institute, Wanju 55365, Republic of Koreakcys0517@kfri.re.kr (Y.-S.C.)

**Keywords:** RAW 2694.7 macrophage, fucoidan, *Sargassum autumnale*, anti-inflammatory

## Abstract

Fucoidans are sulfate-rich polysaccharides with a wide variety of beneficial biological activities. The present study aimed to highlight the anti-inflammatory activity of fucoidan from the brown seaweed *Sargassum autumnale* (SA) against lipopolysaccharide (LPS)-induced RAW 264.7 macrophage cells. Among the isolated fucoidan fractions, the third fraction (SAF3) showed a superior protective effect on LPS-stimulated RAW 264.7 cells. SAF3 inhibits nitric oxide (NO) production and expression of prostaglandin E-2 (PGE2) via downregulation of inducible nitric oxide synthase (iNOS) and cyclooxygenase-2 (COX2) expression in LPS-induced RAW 26.7 cells. SAF3 treatment decreased pro-inflammatory cytokines IL-1β, TNF-α, and IL-6 expression in LPS-induced cells. LPS stimulation activated NF-κB and MAPK signaling cascades in RAW 264.7 cells, while treatment with SAF3 suppressed them in a concentration-dependent manner. Existing outcomes confirm that SAF3 from *S. autumnale* possesses potent anti-inflammatory activity and exhibits good potential for application as a functional food ingredient or for the treatment of inflammation-related disorders.

## 1. Introduction

Brown seaweeds are considered the main natural source of naturally occurring fucoidan [1]. The uniqueness of fucoidan compared to other polysaccharides stems from its abundant L-fucose content and sulfate groups [2]. The structure and compositional properties of fucoidans vary in different species of brown algae [3]. The structural backbone of fucoidan is made up of α-1,3-linked 1-fucopyranose residue repeats or α-1,4-linked 1-fucopyranose residues. In addition, different monosaccharide units such as mannose, galactose, and glucose can be inserted in the fucoidan backbone in place of the repeating units. Fucoidans from several brown seaweeds have been extensively studied in the past decade for their diverse biological activities, including anticoagulant, antitumor, immunomodulatory, and anti-inflammatory effects [4,5,6].

Inflammation is an important response of the immune system that is provoked by various stimuli and conditions, such as pathogens, particulate matter, etc. Inflammation can be acute or chronic and is considered to have the ability to protect from pathogen-induced tissue injury. Chronic inflammation is involved as part of the pathogenesis of conditions such as Alzheimer’s disease, cardiovascular diseases, rheumatoid arthritis, and inflammatory bowel disease [7]. Nevertheless, the inflammatory response is a prime defensive mechanism of the human body and involves the upregulation and activation of many genes. Macrophages get activated on exposure to inflammatory stimuli, such as lipopolysaccharides (LPS). The activated cells produce inflammatory mediators such as nitric oxide (NO), prostaglandin E-2 (PGE2), and pro-inflammatory cytokines, resulting in the activation of signaling pathways such as NF-κB and MAPK [8]. It is reported that the pathogenesis of inflammatory diseases is elicited via the excess production of inflammatory mediators due to the generation of reactive oxygen species (ROS) [9]. The downregulation of pro-inflammatory factors is, therefore, considered a successful therapeutic method for curing inflammation-related diseases [10,11,12]. Fucoidan from brown seaweeds is extensively studied for its anti-inflammatory properties [13]. The ability of fucoidan to boost the efficiency of anti-inflammatory drugs has been studied [14]. Earlier studies have demonstrated the anti-inflammatory effects of fucoidan in a range of experimental contexts, including in vivo and in vitro [15]. However, the beneficial bioactivities of *Sargassum autumnale* have not been extensively studied.

*S. autumnale* is a brown alga found in tropical regions, especially in the intertidal zone of East Asian countries. *Sargassum* spp. are well-known food ingredients in East Asian countries and are also known to possess numerous important and beneficial bioactive compounds [16]. As a food ingredient, it offers a range of nutritional benefits due to its rich composition of essential nutrients, including vitamins, minerals, and antioxidants. Additionally, its unique flavor profile and versatile culinary properties make it an exciting ingredient for creative and nutritious culinary creations [17]. However, *S. autumnale* has not yet been extensively explored in terms of its physical, biological, and biochemical properties as functional ingredients such as fucoidans. A few studies have been conducted on the fucoidan isolated from *S. autumnale* for its antioxidant, antibacterial, antiviral, and anticancer properties [18,19,20]. In our previous study, fucoidan was isolated from *S. autumnale* and found to exhibit excellent antioxidant activity against H_2_O_2_-induced oxidative stress in Vero cells [21]. However, the anti-inflammatory effect of *S. autumnale* sulfated polysaccharides for the treatment of inflammatory diseases has not been explored yet to the best of our knowledge. Thus, the present study was designed as an initial study to assess the potential anti-inflammatory activity and the mechanism of action of *S. autumnale* fucoidan in vitro. The findings may boost the use of the underutilized *S. autumnale* and aid in sustainable development across a range of industries, particularly in the food and functional food sectors, as well as in pharmaceutical development.

## 2. Results

### 2.1. Chemical Composition and Structural Characterization of Isolated S. autumnale Fucoidan Fractions

The chemical composition results are reported in Table 1. Results from the current study revealed that SAP consisted mostly of polysaccharides. The DEAE chromatography resulted in three fucoidan fractions, and the purified polysaccharide fractions contained higher polysaccharide content and sulfate content, with SAF3 possessing the highest levels of sulfates (34.92 ± 0.18%) among the separated fractions. The polysaccharide content showed gradual reduction in the successive fractions while the sulfate content increased. Low protein and polyphenol contents were observed in all the fractions. These results were similar to those obtained in a previous study conducted on the antioxidant effect of sulfated polysaccharides from *Sargassum fulvellum* [22].

Monosaccharide and FTIR analyses of SAF3 were previously reported in our previous study [21]. Monosugar analysis identified that the *S. autumnale* polysaccharides contained five monosaccharides: fucose, rhamnose, galactose, glucose, and xylose. In addition, all the fucoidans contained higher levels of fucose (43.16%) and galactose (34.06%). The FTIR spectra of fucoidans indicated the structural similarity of *S. autumnale* fucoidan with commercial fucoidan. The common IR peak at 1035 cm^−1^ represents the stretching vibration of a glycosidic bridge (C–O–C), while the broad peak at 1220–1270 cm^−1^ represents sulfate groups. The acquired spectra had a low resolution due to the complex, heterogeneous structure of fucoidans.

### 2.2. Evaluation of S. autumnale Fucoidans on Macrophage Cell Viability

**Figure 1:**
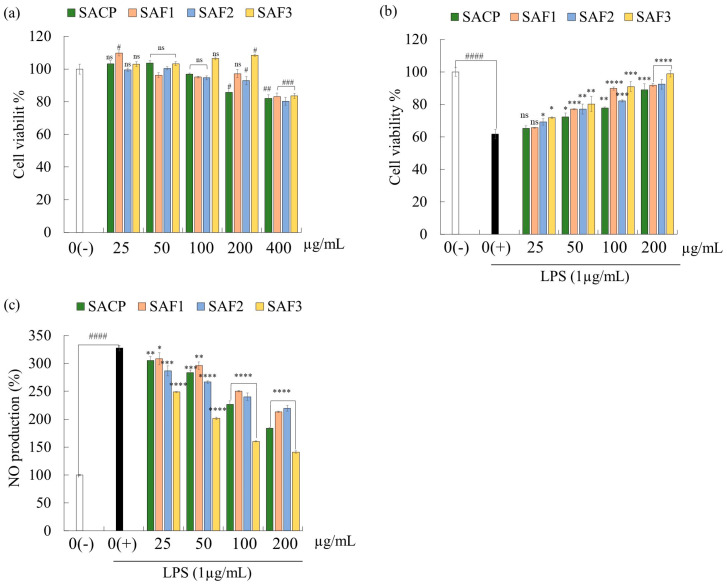
Determination of fucoidan fractions having superior activity. (**a**) sample toxicity, (**b**) protective effect in LPS-induced cells; and (**c**) NO production inhibition in LPS-induced cells. Experiments were performed in triplicate (*n* = 3), and the results are represented as means ± SD. Values are significantly different from LPS-treated group (0(+)) at * *p* < 0.05, ** *p* < 0.01, *** *p* < 0.005, and **** *p* < 0.0001 and from control group (0(−)) at # *p* < 0.05, ^##^ *p* < 0.01, ^###^ *p* < 0.005 and ^####^ *p* < 0.0001. ns denotes not significant.

The effects of the three fractions of fucoidans and crude polysaccharide extract from *S. autumnale* on RAW 264.7 macrophage cells are shown in Figure 1a. Accordingly, with the treatment of SA fucoidans, the cell viability did not decrease significantly at concentrations (25, 50, 100, and 200 μg/mL), suggesting the absence of any adverse effects such as toxic effects of *S. autumnale* extracts on RAW 264.7 cells. Therefore, those concentrations were selected for future experiments. The concentration of 400 μg/mL resulted in decreased cell viability, indicating toxicity to the cells; therefore, it was removed from further experiments. The preliminary concentrations of fucoidan were selected based on previously optimized and published methods [13,23,24].

### 2.3. NO Production Inhibition by S. autumnale Fucoidan Fractions in LPS Stimulated RAW 264.7 Cells

LPS stimulation in RAW 264.7 cells resulted in a significant increase in NO production and a decrease in cell viability compared to non-stimulated cells. Interestingly, fucoidan co-treatment retracted the toxic influence of LPS on macrophage cells, leading to a recovery of cell viability (Figure 1b). NO is known as a key inflammatory mediator essential for preventing inflammation under normal physiological conditions. Nevertheless, elevated NO production under abnormal conditions can cause detrimental effects on tissues [25]. On pre-treatment of cells with *S. autumnale* fucoidans, the high NO production in LPS-stimulated cells decreased in a concentration-dependent manner (Figure 1c). Based on these results, SAF3 was selected for subsequent experiments due to its superior protective activity on LPS-induced RAW 264.7 cells compared to other fractions.

### 2.4. Inhibition of PGE2 and Pro-Inflammatory Cytokines Secretion by SAF3

**Figure 2:**
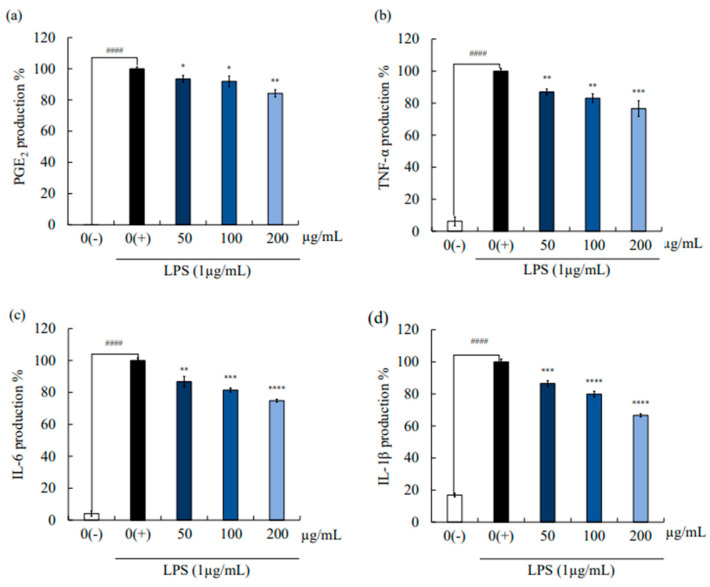
Effect of SAF3 on the inflammatory mediator regulation. SAF3 inhibited the production of (**a**) PGE2, (**b**) TNF-α, (**c**) IL-6, and (**d**) IL-1β pro-inflammatory cytokines. Experiments were performed in triplicate (*n* = 3), and the results are represented as means ± SD. Values are significantly different from the control (LPS treated) group at * *p* < 0.05 and ** *p* < 0.001 compared to untreated group. LPS treated group (0(+)) at * *p* < 0.05, ** *p* < 0.01, *** *p* < 0.005 and **** *p* < 0.0001 and control group (0(−)) at #### *p* < 0.0001.

IL-1β, TNF-α, IL-6-like pro-inflammatory cytokines, and prostaglandin PGE2 play a major role in chronic inflammation due to the upregulation of their production. We tested the inhibitory effect of SAF3 against LPS-induced pro-inflammatory cytokines and PGE2 production using ELISA. As shown in Figure 2, LPS stimulation led to a higher secretion of pro-inflammatory cytokines and PGE2 in RAW 264.7 cells compared to the control group. However, pre-treatment of SAF3 (50, 100, and 200 µg/mL) resulted in a significant reduction in pro-inflammatory cytokine and PGE2 secretion compared to the LPS treated group. According to the results, PGE2 secretion decreased to 84.2% in the 200 µg/mL concentration group from that in the LPS-treated control group. To account for the variability in the data, the error associated with the mean was also determined. The standard deviation was calculated to be ±2.40. In 20 µg/mL concentration group, TNF-α, IL-6, and IL-1β secretions were lowered up to 76.56% ± 4.91, 74.84% ± 0.94, and 66.56% ± 1.24, respectively. Therefore, SAF3 was able to significantly inhibit the expression of pro-inflammatory cytokines and PGE2 during inflammation in RAW 264.7 cells induced by LPS treatment.

### 2.5. Effect of SAF3 on iNOS and COX2 Protein Expression in LPS-Stimulated Cells

**Figure 3:**
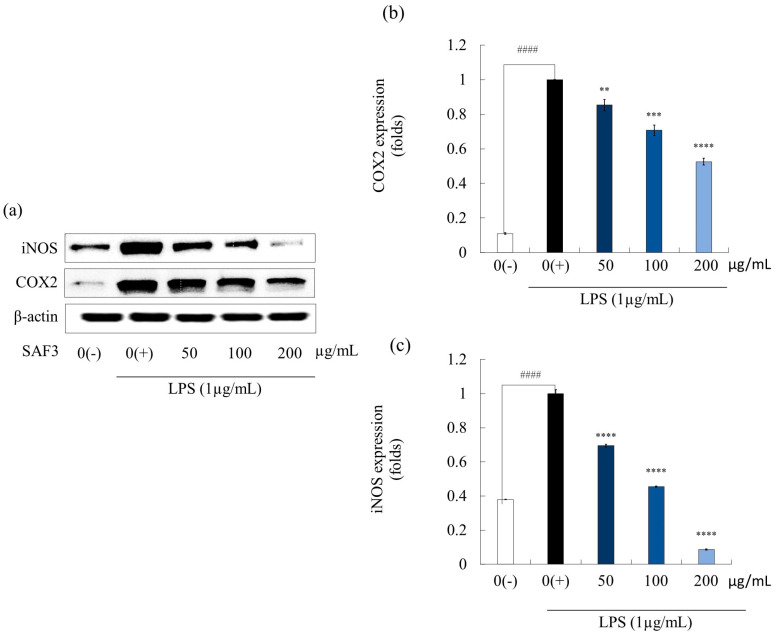
Effects of SAF3 on LPS-induced iNOS and COX2 protein expression in RAW 264.7 cells (**a**) Western blot image showing protein expression, (**b**) COX2, and (**c**) iNOS expression levels were quantified using western blotting after the treatment of LPS-activated macrophages with SAF3. Experiments were carried out in triplicate (*n* = 3), and the results are represented as means ± SD. (*n* = 3). Values are significantly different from the LPS treated group (0(+)) at ** *p* < 0.01, *** *p* < 0.005 and **** *p* < 0.0001 and from control group (0(−)) at #### *p* < 0.0001.

iNOS and COX2 play a vital role in regulating immune responses in cells [26]. The inhibitory activity of SAF3 on iNOS and COX2 expression in LPS-stimulated RAW 264.7 cells was determined using Western blot analysis. The quantitative analysis results indicated that higher expressions of the above-mentioned proteins were obtained from LPS-stimulated cells compared to un-stimulated cells (Figure 3). However, their expression was downregulated when pre-treated with SAF3 in a concentration-dependent manner, demonstrating the anti-inflammatory activity of SAF3. The highest fucoidan concentration resulted in a mean expression of iNOS of 0.087 ± 0.002, while the expression of COX2 was determined to be 0.526 ± 0.019.

### 2.6. SAF3 Downregulated NF-ĸB/MAPK Pathway Proteins Expression

**Figure 4:**
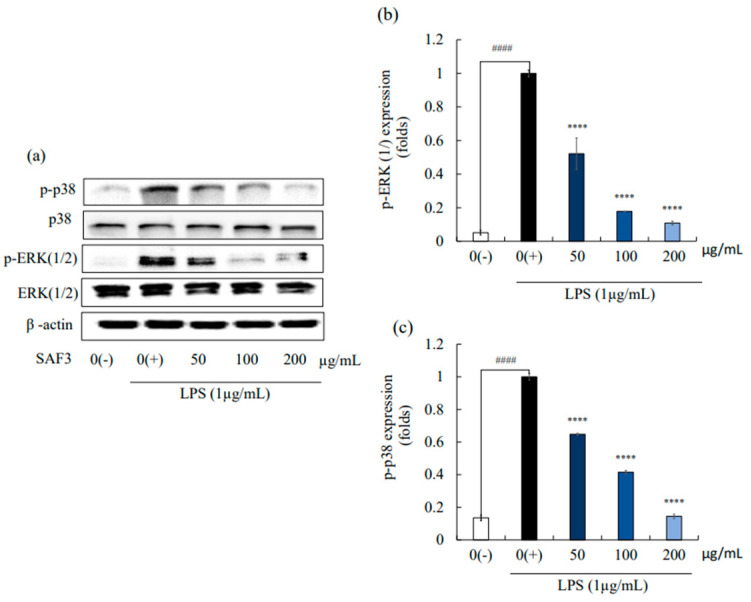
Inhibitory effects of SAF3 on MAPK activation in LPS-stimulated RAW 264.7 cells. (**a**) Western blot image showing protein expression; (**b**) p-ERK; and (**c**) p-p38 expression in RAW 264.7 cells. Experiments were performed in triplicate (*n* = 3), and the results are represented as means ± SD. Values are significantly different from LPS treated group (0(+)) at **** *p* < 0.0001 and from control group (0(−)) at #### *p* < 0.0001.

**Figure 5:**
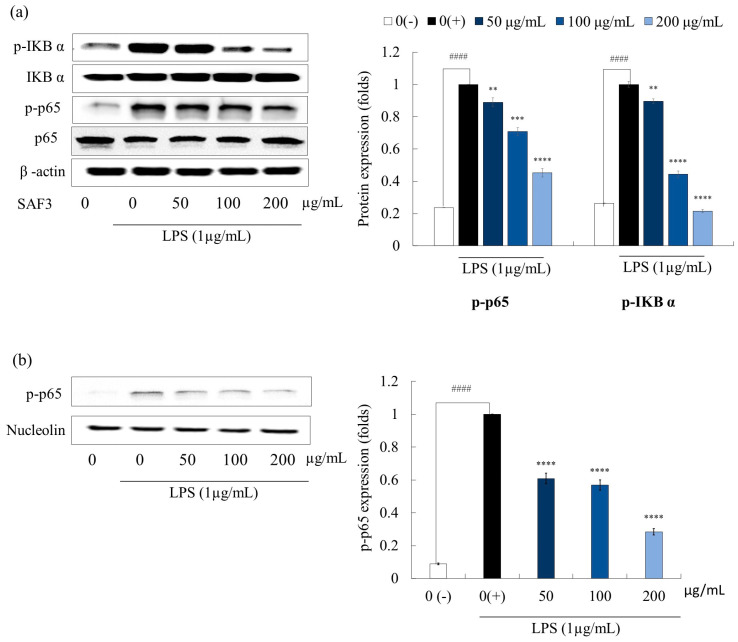
Inhibitory effects of SAF3 on NF-ĸB activation in LPS-stimulated RAW 264.7 cells. (**a**) p-p65 and p-IKB⍺ expression in cytosol, and (**b**) p-p65 expression in nucleus. Experiments were performed in triplicate (*n* = 3), and the results are represented as means ± SD. Values are significantly different from LPS treated group (0(+)) at ** *p* < 0.01, *** *p* < 0.005 and **** *p* < 0.0001 and from control group (0(−)) at #### *p* < 0.0001.

MAPK and NF-ĸB signaling cascades exist in eukaryotic cells and play a vital role in a wide variety of cellular functions. MAPK phosphorylation inhibition is a potential approach to curing inflammation-related diseases [27]. The effect of SAF3 on the regulation of the expression of these pathway proteins was analyzed using Western blotting. LPS stimulation initiated the activation of MAPK (Figure 4) and NF-ĸB (Figure 5) cascades. Stimulation of LPS resulted in the significant phosphorylation of MAPK proteins (*p* < 0.05), such as p38 and ERK; the band intensity of these proteins was higher in the un-stimulated group. Further, treatment of SAF3 interestingly downregulated this elevated expression of the phosphorylation forms of p38 and ERK.

In unstimulated cells, NF-ĸB, which is located in the cytoplasm, combines with IKBα-like inhibitory proteins and forms an inactive form (p50-p65-IĸB). LPS stimulation of cells resulted in phosphorylation of the inactive former, allowing IĸBα to be released and translocated into the nucleus [28]. According to the results obtained in our study, the phosphorylation of p65 and IKBα was enhanced by LPS stimulation in the cytosol and nucleus compared to the control group, respectively (Figure 5a,b). However, pre-incubation with SAF3 significantly reduced the phosphorylation of NF-ĸB proteins in a concentration-dependent manner. The mean expressions of p-p65 and p-IKBα in the cytosol were found to be 0.453 ± 0.026 and 0.215 ± 0.010 at the maximum fucoidan concentration, respectively. Moreover, the nuclear expression of p-p65 was also downregulated concentration-dependently. This solidifies the ability of SAF3 to downregulate the nuclear translocation of p65 in the NF-κB signaling pathway. These results showed that SAF3 has good anti-inflammatory activity due to the suppression of MAPK and NF-κB phosphorylation in LPS-induced RAW264.7 cells.

## 3. Discussion

Fucoidans represent a group of fucose-rich sulfated polysaccharides found especially in brown algae. They are mainly made of fucose and sulfate groups and contain other molecules such as uronic acid, xylose, mannose, and galactose. Fucoidans are extensively studied as they exhibit a number of biological activities such as anti-inflammatory, anti-viral, antioxidant, anti-coagulant, and antitumor activities [29,30,31,32]. Experimental evidence suggests that fucoidan possesses anti-inflammatory activity and has excellent potential applications as an anti-inflammatory agent. The identification and characterization of seaweed fucoidan provide great opportunities for their development and application as functional foods and medicines for the treatment of various diseases with fewer side effects. However, the anti-inflammatory potential of fucoidan from *S. autumnale* has not yet been comprehensively studied. Therefore, in the current study, we evaluated and demonstrated the in vitro anti-inflammatory activity of fucoidan extracted from *Sargassum autumnale*.

Enzyme-assisted extraction is one of the most popular methods for extracting such important bioactive compounds from seaweed. This method is widely used in industrial extraction owing to its high effectiveness, low pollution, and low production costs [33]. In this study, enzyme-assisted extraction was used to augment the bioactive characteristics of fucoidan; the crude polysaccharide was further purified using DEAE-cellulose column chromatography, which yielded three fractions. The pooled three fractions were designated as SAF1, SAF2, and SAF3. According to the preliminary experiments, such as its cytoprotective effect and ability to downregulate NO production properties in LPS-induced macrophage cells, SAF3 showed superior activity among the other fractions. Moreover, according to the compositional analysis, SAF3 contained higher sulfate and fucose content, which is important for good anti-inflammatory activities [13]. The inclusion of differing quantities of sulfate groups, polyphenols, proteins, the structural placement of sulfated groups, structure, and molecular weight affect the biological activities of fucoidan [34]. According to several studies, having more polyphenols increases their anti-inflammatory effects [35]. However, in our investigation, the compositional analysis revealed minimal levels of proteins and polyphenols and substantially greater concentrations of polysaccharides and sulfates. Therefore, the anti-inflammatory activity of SAF3 can be identified as being mainly due to its sulfate and polysaccharide content. The lower polyphenol content in SAF3 is due to the removal of polyphenols as lipids with depigmentation. The higher sulfate and polysaccharide quantities were due to the active anion exchange column system [36]. Therefore, SAF3 was selected for further study. The FTIR analysis showed that SAF3 has a similar structure to commercial fucoidan. However, it is reported that fucoidan derived from different seaweeds exhibits significantly different levels of bioactivity. The variation in bioactivities may arise from several factors related to the seaweed’s species, geographical location, environmental conditions, extraction methods, and molecular structure. According to previously published reports, different seaweed species contain distinct compositions of fucoidan, with variations in the ratio and types of monosaccharides, sulfate content, and molecular weight. These differences can influence the bioactivity of fucoidan and its interactions with biological systems [37]. Furthermore, the extraction method employed in the present study to isolate fucoidan may also affect its bioactivity. As reported in early studies, extraction techniques such as solvent selection, temperature, and time duration can impact the preservation of bioactive compounds and the structural integrity of fucoidan with varying degrees of bioactivity [38]. Therefore, although fucoidan from different seaweeds may exhibit identical or higher similarity in FTIR structure profiles, their bioactivities can differ significantly. Therefore, it is crucial to study and evaluate the bioactivity of fucoidan from *S. autumnale* to understand its potential application in different fields.

NO is a key inflammatory mediator that induces inflammation and plays a major role in pathogenesis and infectious disorders [39]. LPS exposure has been proven to increase the production and release of NO in macrophages, with the increased production of NO in cells potentially causing tissue damage [25]. Therefore, NO inhibition is important in therapeutic interventions for the treatment of inflammatory diseases [40]. The anti-inflammatory potential of fucoidan isolated from various brown seaweeds has been demonstrated in previous studies [31,34,41]. Apart from NO, PGE2 and pro-inflammatory cytokine production were also increased due to the LPS stimulation in macrophage cells. PGE2 is a principal inflammatory mediator whose production occurs in cells in response to external stimuli. Increased release of PGE2 can cause unfavorable inflammatory responses, which lead to tissue damage [42]. Immune cells such as neutrophils, macrophages, and eosinophils are also involved in the pathogenesis of inflammation through the production of inflammatory cytokines such as TNF-α, IL-6, and IL-1β. Abnormal production of the above-mentioned cytokines and PGE2 may result in the development of inflammatory lesions and pose the threat of inducing apoptosis in the cells [43]. Therefore, inhibition of their excess production is important in controlling inflammation [44].

According to our study, the LPS treatment resulted in increased release of NO, PGE2, and pro-inflammatory cytokines in RAW 264.7 cells, and the production of these compounds was significantly down-regulated by co-treatment with SAF3 concentration-dependently. Our findings are consistent with the results obtained from studies conducted on the anti-inflammatory properties of sulfated polysaccharides from *S. swartzii*, *C. minima*, and *E. cava* [5,23,45]. In addition, iNOS and COX2 expression during the inflammatory response result in NO and PGE2 production. Previous studies have demonstrated using Western blot analysis that sulfated polysaccharides such as fucoidan derived from seaweeds inhibit iNOS and COX2-like mediators in stimulated cells [10]. The key contribution to the production of NO is considered to be arginine oxidation by nitric oxide synthase (NOS), whereas PGE2 synthesis is due to the involvement of COX2 [46]. In agreement with the previous reports, COX2 and iNOS expression were repressed in our study on the treatment with SAF3 in LPS-stimulated RAW 264.7 cells. Similar results were obtained in a study on the anti-inflammatory effect of *S. swartzii* in LPS-induced RAW 264 cells [23].

It is reported that activation of complex signaling pathways such as NF-κB/MAPK results in inflammatory responses [10,47,48]. Therefore, the effect of SAF3 samples on the downstream signaling of these pathways was further examined. The NF-κB transcription factor family contains c-Rel, RelB, p50, p65, and p52 proteins. The NF-κB transcription factor resides in the cytosol under normal conditions, and it is translocated into the nucleus in its phosphorylated form due to the stimulation of macrophages [48,49]. This results in the transcription of inflammatory-related genes such as iNOS and COX2, as well as genes encoding pro-inflammatory cytokines [50]. This study revealed that the LPS stimulation promoted IKBα and p65 phosphorylation, consequently activating the NF-κB pathway. Nonetheless, down-regulation of the phosphorylation of NF-κB transcription factors and their nuclear translocation was achieved by concentration-dependent treatment with fucoidan. These results were also observed downstream of the MAPK signaling cascade. MAPK plays a major role in the release of inflammatory chemokines and the activation of pro-inflammatory cytokines [51,52]. Among the three important MAPKs (p38, JNK, and ERK (1/2)), p38 is involved in regulating the synthesis of inflammatory regulators [53,54]. With LPS-stimulation, the phosphorylation of p38 and ERK (1/2) was observed to increase, which was downregulated with the treatment with fucoidan (SAF3). Therefore, inhibition of protein expression linked with NF-κB/MAPK signaling can be considered a promising target for the treatment of inflammation-related diseases. Our study findings corroborate those of other research on the anti-inflammatory properties of fucoidan isolated from *E. maxima*, *C. minima*, and *S. horneri* [41]. The current study improves our understanding of cellular mechanisms related to LPS-induced inflammation. Overall, the observations obtained from the current study verify the potential of SAF3 against chronic inflammatory conditions. This also encourages further studies to confirm the relationship between the anti-inflammatory activity of *S. autumnale* fucoidan and TLR-mediated NF-κB/MAPK signaling pathways using pharmacological inhibitors.

This manuscript represents a pioneering research article focused on investigating the anti-inflammatory activity of fucoidan derived from *S. autumnale.* It stands as the first of its kind to comprehensively explore the potential therapeutic effects of fucoidan specifically sourced from *S. autumnale* in the context of inflammation. By filling this crucial research gap, the obtained results of the study have paved the way for a deeper understanding of the anti-inflammatory properties of *S. autumnale* fucoidan and its potential applications in the field of medicine. This highlights the importance of studying the unique characteristics and bioactivity of fucoidan from different sources. The findings presented will serve as a valuable foundation for future studies and inspire further exploration of the therapeutic potential of *S. autumnale* fucoidan in the treatment and management of inflammatory conditions.

## 4. Materials and Methods

### 4.1. Chemicals and Reagents

Protomax enzyme, Polysaccharide standards (fucoidan), and 3-(4,5-dimethylthiazol-2 yl)-2,5-diphenyltetrazolium bromide (MTT) were obtained from Sigma-Aldrich (St. Louis, MO, USA). The murine macrophage cell line RAW 264.7 was purchased from the American Type Culture Collection (Rockville, MD, USA). Dulbecco’s modified Eagle’s medium (DMEM), fetal bovine serum (FBS), and penicillin-streptomycin were purchased from Gibco/BRL (Burlington, ON, Canada). Enzyme-linked immunosorbent assay kits for PGE2, IL-6, and IL-1β were acquired from R & D Systems Inc. (Minneapolis, MN, USA). Carbohydrate-degrading enzymes were donated by Novozymes (China) Biotechnology Co., Ltd. (Tianjin, China). Ethanol, 2-propanol, the BCA protein assay kit, and chloroform were purchased from Sigma–Aldrich. Primary and secondary antibodies were purchased from Santa Cruz Biotechnology (Dallas, TX, USA). An enhanced chemiluminescence reagent was obtained from Amersham (Arlington Heights, IL, USA). All other chemicals and solvents used in this study were of analytical grade.

### 4.2. Sample Collection, Enzyme Assisted Extraction, and Enrichment of Fucoidan

*Sargassum autumnale* was collected from the coast of Jeju Island, Republic of Korea, in March 2018. The seaweed was cleaned thoroughly with water for the removal of epiphytes and other impurities and dried using a Goodle dryer [18]. Dried seaweed was then ground into a fine powder. The powder (10 g) was dissolved in 1 L of distilled water (DW) and treated with Protomax (Omax, Kent, WA, USA) (pH 6.0, 40 °C) for 24 h. The enzyme was then inactivated by heat treatment at 100 °C for 10 min, and the pH was adjusted to 7 (SAP). Precipitation of polysaccharides was done by addition of 95% ethanol and stored at 4 °C for 8 h for the precipitation of polysaccharides. Precipitated polysaccharides were recovered by centrifugation and washed with 95% ethanol by homogenizing and centrifugation. Finally, a *S. autumnale* crude polysaccharide (SACP) sample was freeze-dried and used for further experiments.

### 4.3. Anion Exchange Chromatography for Separation and Purification of Fucoidan

A crude polysaccharide solution was prepared in sodium acetate buffer and loaded onto a DEAE cellulose column [21]. Fucoidan fractions were separated using ion-exchange chromatography by eluting with a linear gradient of sodium chloride (50–2000 mM). Separated fucoidan fractions (SAF1, SAF2, and SAF3) were collected, dialyzed, and finally lyophilized.

### 4.4. Chemical Composition Analysis

Estimates of the polysaccharide, protein, polyphenol, and sulfate contents of the fractions were made using accepted techniques described in publications by the Association of Official Agricultural Chemists [21]. In order to ascertain the polysaccharide and polyphenol contents, respectively, the phenol-sulfuric and Folin–Ciocalteu procedures were applied. The amount of protein was determined using Lowry protein assays, while the amount of sulfate was determined using the barium sulfate precipitation technique.

### 4.5. Cell Culture

Murine macrophage cell line RAW 264.7 was purchased from the American Type Culture Collection (ATCC) (TIB-71) and maintained in DMEM with 10% FBS and 1% penicillin/streptomycin mixture at 37 °C in a 5% CO_2_ humidified atmosphere. Cells were subjected to a periodic subculture, and cells showing exponential growth were used for further experiments. LPS stock solution was prepared using PBS solution, and it was diluted when necessary. *S. autumnale* extracts were dissolved in PBS solutions and diluted to prepare working concentrations.

### 4.6. Cell Viability and NO Production

Cell viability was evaluated by an MTT assay to measure the cytotoxicity of samples [13,24]. In brief, RAW 264.7 cells were seeded in a 96-well plate and incubated for 24 h in a CO_2_ incubator prior to the treatment of SACP and *S. autumnale* fucoidan (25, 50, 100, 200, and 400 μg/mL). The sample concentrations were selected based on previously published results [55]. The MTT assay was carried out after 24 h incubation. For the analysis of the cytoprotective effect of samples against LPS stimulation, *S. autumnale* extracts were applied to the cells (25, 50, 100, and 200 μg/mL), and after 1 h, cells were stimulated with LPS (final concentration 1 μg/mL). Following the incubation, MTT reagent was added to the wells and incubated for another 2 h in a humidified incubator. Finally, MTT was removed after 2 h, and 100 μL of DMSO solution was added; absorbance was measured at 570 nm after 10 min [10].

The Griess assay was employed to determine the ability of *S. autumnale* fucoidan to inhibit NO production in LPS-stimulated RAW 264.7 cells. Briefly, the seeded cells were incubated for 24 h prior to the application of *S. autumnale* extracts at different concentrations. After 24 h of stimulation with LPS, the cell culture medium and Griess reagent were mixed in equal amounts, and absorbance was measured at 540 nm after 10 min on an ELISA plate reader (BioTek Instruments, Inc., Winooski, VT, USA). The fucoidan fraction SAF3 showed excellent NO inhibition activity and cytoprotective activity against LPS. Therefore, it was selected for further experiments [13,31].

### 4.7. Assessment of PGE2 and Pro-Inflammatory Cytokines Expression Levels

For the assessment of prostaglandin and pro-inflammatory cytokine levels, we cultured RAW 264.7 cells were treated with different concentrations of SAF3 (50, 100, and 200 μg/mL). After 1 h, LPS was applied to the cells and further incubated for 24 h. PGE2, IL-6, IL-1β, and TNF-α were quantified in the cell-free supernatant with the help of competitive enzyme immunoassay kits (R & D Systems Inc.) [41].

### 4.8. Western Blot Analysis

The effect of SAF3 on the expression levels of iNOS, COX2, NF-κB, and MAPK pathway proteins was analyzed using Western blotting [24]. In brief, seeded RAW 264.7 cells were treated with SAF3 (50, 100, and 200 μg/mL) for 2 h, followed by LPS stimulation of the cells for 24 h. Harvested cells were processed for protein extraction, and the BCA protein assay kit was used to determine protein concentration. The proteins were then separated on a 12% SDS-PAGE gel under denaturing conditions and transferred onto a nitrocellulose membrane. The membrane was blocked with 5% skim milk prior to the incubation with primary antibodies. Membranes were incubated with primary antibodies overnight at 4 °C, followed by incubation with secondary antibodies for 2 h at room temperature. Chemiluminescent substrate (Cyanagen Srl, Bologna, Italy) was used for the development of signals, and the bands were photographed using the FUSION SOLO Vilber Lourmat system. The ImageJ program (version 1.50i, Wayne Rasband, National Institute of Health, Bethesda, ML, USA) was aided in the quantification of band intensities [56].

### 4.9. Statistical Analysis

All the cell experiments were triplicated, and all data are expressed as means ± SD. Significant differences among data values were determined with the Kruskal–Wallis test, Mann–Whitney U test. All statistical analyses were performed using the GraphPad Prism 9 statistical analysis package (GraphPad Software Inc., San Diego, CA, USA). A *p*-value < 0.05 is considered significant.

## 5. Conclusions

In summary, this study reveals the potential anti-inflammatory activity of purified fucoidan (SAF3) from *S. autumnale* against LPS-induced inflammation. SAF3 significantly inhibited the release of NO, PGE2, and pro-inflammatory cytokines such as TNF-α, IL-6, and IL-1β in RAW 264.7 macrophage cells. The study also exhibited that the anti-inflammatory activity was related to the downregulation of iNOS and COX2 and signaling pathways such as NF-κB and MAPK. Thus, the fucoidan isolated from *S. autumnale* can be developed as a broad-spectrum anti-inflammatory drug or other health product.

Furthermore, the research findings provide an understanding of the continuous exploitation of compounds from marine algae for the prevention and mediation of inflammatory diseases. Further in vivo experiments are needed to test the anti-inflammatory properties of *S. autumnale* fucoidan before it can be developed into a human-consumable product.

## Figures and Tables

**Table 1:** Chemical composition of purified fucoidan from *S. autumnale*.

Sample	Polysaccharide%	Protein%	Polyphenol%	Sulfate%
SAP	30.25 ± 0.44	15.14 ± 0.21	10.3 ± 0.14	7.81 ± 0.24
SACP	59.34 ± 1.01	9.19 ± 0.83	8.00 ± 0.01	11.07 ± 2.09
SAF1	34.28 ± 0.50	7.99 ± 0.38	3.58 ± 0.36	21.44 ± 0.50
SAF2	24.55 ± 0.12	5.41 ± 0.14	2.91 ± 0.32	25.88 ± 0.51
SAF3	18.04 ± 0.98	2.01 ± 0.08	1.95 ± 0.18	34.92 ± 0.18

## Data Availability

Data will be made available upon request.
